# Flavivirus Capsid Proteins Inhibit the Interferon Response

**DOI:** 10.3390/v14050968

**Published:** 2022-05-05

**Authors:** Adriana M. Airo, Alberto Felix-Lopez, Valeria Mancinelli, Danyel Evseev, Joaquin Lopez-Orozco, Kathy Shire, Patrick Paszkowski, Lori Frappier, Katharine E. Magor, Tom C. Hobman

**Affiliations:** 1 Department of Medical Microbiology & Immunology, University of Alberta, Edmonton, AB T6G 2E1, Canada; airo@ualberta.ca (A.M.A.); felixlop@ualberta.ca (A.F.-L.); ppaszkow@ualberta.ca (P.P.); 2 Department of Cell Biology, University of Alberta, Edmonton, AB T6G 2H7, Canada; valeriam@ualberta.ca; 3 Department of Biological Sciences, University of Alberta, Edmonton, AB T6G 2E9, Canada; evseev@ualberta.ca (D.E.); kmagor@ualberta.ca (K.E.M.); 4 High Content Analysis Core, University of Alberta, Edmonton, AB T6G 2E1, Canada; lopezoro@ualberta.ca; 5 Department of Molecular Genetics, University of Toronto, Toronto, ON M5G 1M1, Canada; kathy.shire@utoronto.ca (K.S.); lori.frappier@utoronto.ca (L.F.); 6 Li Ka Shing Institute of Virology, University of Alberta, Edmonton, AB T6G 2E1, Canada

**Keywords:** flaviviruses, global transcription, capsid protein, interferon response, TRIM25, Zika virus

## Abstract

Zika virus (ZIKV) establishes persistent infections in multiple human tissues, a phenomenon that likely plays a role in its ability to cause congenital birth defects and neurological disease. Multiple nonstructural proteins encoded by ZIKV, in particular NS5, are known to suppress the interferon (IFN) response by attacking different steps in this critical antiviral pathway. Less well known are the potential roles of structural proteins in affecting the host immune response during ZIKV infection. Capsid proteins of flaviviruses are of particular interest because a pool of these viral proteins is targeted to the nuclei during infection and, as such, they have the potential to affect host cell gene expression. In this study, RNA-seq analyses revealed that capsid proteins from six different flaviviruses suppress expression of type I IFN and IFN-stimulated genes. Subsequent interactome and in vitro ubiquitination assays showed that ZIKV capsid protein binds to and prevents activating ubiquitination of RIG-I CARD domains by TRIM25, a host factor that is important for the induction arm of the IFN response. The other flavivirus capsid proteins also interacted with TRIM25, suggesting that these viral proteins may attenuate antiviral signaling pathways at very early stages of infection, potentially even before nonstructural proteins are produced.

## 1. Introduction

The genus *flavivirus* is comprised of over 70 members including medically important mosquito-transmitted viruses such as Dengue virus (DENV), Japanese encephalitis virus (JEV), yellow fever virus (YFV), and Zika virus (ZIKV). ZIKV was originally isolated from a sentinel rhesus monkey in 1947 in Uganda [1] and for approximately 60 years, it silently circulated in Africa and Asia [2]. The first known epidemic of ZIKV occurred in 2007 on Yap island in the Federated States of Micronesia [3] followed by epidemics in the Pacific Islands [4]. ZIKV began circulating in Brazil in late 2013 or early 2014 [5,6] before rapidly spreading to other countries in the Americas. Although infection is often asymptomatic, ZIKV infection is now known to be associated with microcephaly and other fetal development abnormalities in fetuses [7] as well as the neurological disorder Guillain-Barré syndrome [8]. Given the unprecedented rise of flavivirus infections in the Americas, it is important to further understand the mechanisms by which ZIKV and other flaviviruses affect host cell signaling pathways to facilitate replication.

The type I interferon (IFN) response is critical for controlling flavivirus infections, as evidenced by the fact that mice which lack the ability to induce expression of IFN-α/β or initiate downstream signaling, are more susceptible to infection and severe disease [9,10,11,12,13,14]. Host cells can sense viral pathogens through pattern recognition receptors present on the cell surface and in intracellular compartments. These include toll-like receptors, retinoic acid-inducible gene I-like receptors, cytosolic DNA sensors, and the nucleotide oligomerization domain-like receptors [15]. The genomic RNA of flaviviruses can be recognized by the cytoplasmic retinoic acid-inducible gene I-like receptor RIG-I [16], an RNA helicase that contains two caspase-activation and recruitment (CARD) domains at the N-terminal region, a DExD/H box helicase domain, and a C-terminal domain [17,18]. Binding of the helicase and C-terminal domains to viral RNA induces a conformational change that exposes the CARD domains. Following dephosphorylation of the CARD domains by PP1-α/γ [19], RIG-I is polyubiquitinated at lysine 172 by the E3 ubiquitin ligase tripartite motif-containing protein 25 (TRIM25) [20]. Polyubiquitination of RIG-I is required to initiate a signaling cascade leading to phosphorylation of transcription factors IRF3 and NF-kB, and eventually IFN production and expression of antiviral genes [20,21].

Flaviviruses have positive single-stranded RNA genomes of approximately 11 kb that encode three structural proteins: capsid, the precursor membrane (prM), the envelope protein; and seven nonstructural proteins: NS1, NS2A, NS2B, NS3, NS4A, NS4B and NS5. Nonstructural proteins of flaviviruses have been shown to interfere with the induction and/or signaling phases of the type I IFN response [22,23,24,25,26,27,28]. Whether flavivirus structural proteins affect the IFN response has not been studied extensively, but we and others have shown that the capsid protein, whose main role is to encapsulate the viral genome, plays additional nonstructural roles [29,30,31,32]. Although flavivirus assembly occurs on the endoplasmic reticulum, a large pool of the capsid protein localizes to the nucleus during infection (reviewed in [33]). The role of flavivirus capsid proteins in the nucleus during infection is largely unknown but by binding to core histones [34], these viral proteins could affect host cell gene expression. Aggregate transcriptomic data from cells expressing capsid proteins from six different flaviviruses revealed that expression of interferon-stimulated genes (ISGs) was dramatically suppressed. Subsequent analyses showed that the ZIKV capsid protein, and likely other flavivirus capsid proteins, subverts the type I IFN response in part by interacting with TRIM25, thereby blocking the ability of this E3 ubiquitin ligase to activate RIG-I and downstream IFN signaling.

## 2. Materials and Methods

### 2.1. Mammalian Cell Culture and Virus Infections

Human alveolar epithelial carcinoma (A549) and Vero cells were obtained from the American Type Culture Collection (ATCC) and cultured in DMEM supplemented with 10% fetal bovine serum (FBS) (GIBCO). Cells were maintained at 37 °C in a 5% CO_2_ atmosphere. The Asian ZIKV strain isolated in Puerto Rico (PRVABC-59) was kindly provided by Dr. Dave Safronetz (Public Health Agency of Canada, Winnipeg, Canada) and was propagated in *Aedes albopictus* C6/36 cells and then titered on Vero cells.

### 2.2. Production of Lentivirus and Transduction of Cells

Recombinant lentiviruses encoding different myc-tagged flavivirus capsids and AcGFP were harvested from transfected HEK293T cells as described earlier by ourselves [35]. Transduction of A549 cells was performed in DMEM with 3% FBS and 5 μg/mL polybrene by spin inoculation (1200× *g* for 1 h at 37 °C).

### 2.3. Sample Preparation for RNA-Sequencing

A549 cells (1 × 10^5^ cells per well) were seeded into 6-well plates and transduced as previously described using a multiplicity of transduction (MOT) of 10. Forty-eight hours post-transduction cells were collected for flow cytometry analysis, or were pelleted from duplicate wells, washed with phosphate-buffered saline (PBS) 137 mM NaCl, 2.7 mM KCl, 8 mM Na_2_HPO_4_ (pH 7.4) and stored at minus 80 °C for subsequent RNA extraction. Flow cytometry analysis of lentivirus transduced cells confirmed that all samples were approximately 90% AcGFP-positive and thus expressing flavivirus capsid proteins. RNeasy mini kit (QIAGEN) was used for total RNA isolation from frozen cell pellets with the additional DNase treatment included in the sample preparation. RNA concentrations were quantified by Qubit (Invitrogen) and the quality/integrity of the RNA samples was verified using a Bioanalyzer (Agilent Technologies) prior to sequencing. All samples had RNA integrity numbers (RIN) > 8.0.

### 2.4. Ion Torrent Next-Generation Sequencing

Genome-wide targeted amplicon amplification was performed using the Ion AmpliSeq™ Transcriptome Human Gene Expression Kit (Thermo-Fisher, Ottawa, ON, Canada). Total RNA (100 ng) was used to prepare the libraries as per manufacturer’s protocol and described elsewhere [36]. Prepared libraries were quantified using the Ion Library TaqMan Quantitation kit (Thermo-Fisher, Ottawa, ON, Canada) and then the RNA concentration was adjusted to 100 pM. An Ion Chef instrument (Thermo-Fisher, Ottawa, ON, Canada) was used for loading of Ion 540 chips (Thermo-Fisher, Ottawa, ON, Canada) after which sequencing was performed on an Ion S5 sequencer (Thermo-Fisher, Ottawa, ON, Canada).

### 2.5. Differential Gene Expression (DEG) Analysis

The differentially expressed genes (DEGs) of transduced cells expressing different flavivivirus capsid proteins (DENV, JEV, MVEV, SLEV, YFV, ZIKV) samples were compared against cells transduced with lentivirus encoding AcGFP only (Control) samples using Partek^®^ Flow Software. Briefly, the reads obtained from an Ion S5 sequencer were aligned against the human genome GRCH37 (hg19) using STAR aligner. The reads from capsid-expressing cells were normalized against those from cells expressing AcGFP only using RPKM (reads per kilobase of exon per million) normalization method. The differential expression was calculated using DESeq2. The cut-off values for highly confident hits were determined using a *p*-value of ≤0.01 and a fold change ≤2 or ≥2. Gene ontology of the highly differentially expressed genes was analyzed using the online server GOrilla [37], DAVID [38,39] and GO Enrichment Analysis (Geneotology.org) [40,41,42].

### 2.6. RNA Extraction and qRT-PCR

RNA isolation was performed using the RNA NucleoSpin kit as per manufacturer’s instructions (Macherey-Nagel) after which complementary DNA (cDNA) was generated using qSCript cDNA SuperMix (Quanta, Gaithersburg, MD, USA). Quantitative PCR was performed using PerfeCTa SYBR Green SuperMix with low ROX (Quanta Biosciences) using a Stratagene Mx3005P real time PCR instrument or a Bio-Rad CFX96 Touch Real-Time PCR instrument. Relative RNA transcript levels were determined using the delta Ct method using β-actin as the housekeeping gene. A list of primers can be found in Supplementary Table S1.

### 2.7. Validation of RNA-Seq

Complementary DNA (cDNA) was generated from RNA isolated from the same samples that were used for RNA-sequencing, and quantitative PCR was performed as previously above. Primer sequences from eight selected genes used for the Ion AmpliSeq Transcriptome Human Gene Expression Kit (Thermo-Fisher, Ottawa, ON, Canada) were obtained from Integrated DNA Technologies (IDT). RNA expression levels were determined using the delta Ct method using β-actin as the housekeeping gene. The fold-change value obtained by RNA-sequencing was plotted against the fold change value obtained by qRT-PCR and the regression line was plotted.

### 2.8. Immunoblotting

A549 or HEK293T cells were collected at designated time points, washed once in PBS before lysis in cold lysis buffer (5 M NaCL, 0.5 M EDTA pH 8.0, 1 M Tris-HCl pH 8.0, 1% Triton X-100, 10% sodium deoxycholate, 10% SDS) or 150 mM NaCl, 25 mM Tris-HCl (pH 7.6), 1% NP40, 1% sodium deoxycholate and 0.1% SDS supplemented with cOmplete™ mini EDTA-free Protease inhibitor cocktail (Sigma, St. Louis, MO, USA) and PhosSTOP phosphatase inhibitor cocktail (Roche). Cell lysates were incubated for 20 min with rotation at 4 °C and subsequently cleared by centrifugation for 15 min at 14,000 rpm at 4 °C in a microfuge. Laemmli sample (2X) buffer (0.125 Tris-HCl pH 6.8, 4% SDS, 20% glycerol, 10% β-mercaptoethanol, 0.004% bromophenol blue) was added to cleared lysates then heated at 95 °C for 5 min prior to separation by polyacrylamide gel electrophoresis. Proteins were transferred to polyvinylidene fluroride membranes which were then blocked for at least one hour in PBS containing 5% bovine serum albumin (BSA) prior to incubation with primary antibodies diluted in the solution. Primary antibodies were detected by incubating the membranes with fluorescent secondary antibodies in PBS containing 5% BSA for 1 h. Proteins were visualized using a LiCor Odyssey detection system (LiCor Biosciences, Lincoln, Dearborn, MI, USA).

### 2.9. Antibodies

The following antibodies were obtained from the indicated sources: Goat anti-GFP, rabbit anti-TRIM25/EFP, mouse anti-actin from Abcam; mouse anti-myc 9e10 antibody from the American Type Culture collection; rabbit anti-phospho-IRF-3 (Ser396) (4D4G), rabbit anti-IRF-3 (D6I4C), rabbit anti-FLAG from Cell Signaling Technology; rabbit anti-ZIKV capsid from GeneTex; mouse anti-HA clone HA-7 from Sigma-Aldrich. The mouse monoclonal anti-ZIKV NS1 8F1 was produced in house using ProSci Inc.

### 2.10. Flow Cytometry Analysis of ZIKV-Infected Cells

A549 cells (2 × 10^5^ cells per well) were seeded into 6-well plates and transduced the next day with lentivirus encoding AcGFP alone or AcGFP and ZIKV capsid at a multiplicity of transduction of 3 by spin inoculation as described above. The next day cells were infected with ZIKV strain PRVABC-59 at a multiplicity of infection of 5. Cells were collected 48 h post-infection by trypsinization and then fixed in PBS containing 4% paraformaldehyde for a minimum of 1 h at 4 °C, followed by washing with PBS. Cells were permeabilized and blocked using PBS containing 0.2% Triton X-100 and 10% FBS for 10 min on ice. After washing in PBS, cells were incubated with mouse anti-ZIKV NS1 8F1 antibody for 1 h at room temperature. Cells were then washed once with PBS and then incubated with donkey anti-mouse Alexa Fluor 647 for 30 min. Following a wash in PBS, cells were resuspended in PBS containing 1% BSA and 5 mM EDTA before analysis using a LSRFortessa X20 SORP flow cytometer (BD Biosciences, East Rutherford, NJ, USA).

### 2.11. Generation of Stably Transduced Cell Lines

A549 cells were transduced with lentiviruses encoding AcGFP or AcGFP and ZIKV capsid. Twenty-four hours post-transduction AcGFP-positive cells were sorted using a FACSAria III cell sorter (BD Biosciences) and plated at 1 cell/well into 96-well plates. Cells were cultured under observation for approximately 3 weeks, after which ten colonies from AcGFP or AcGFP-ZIKV capsid transduced populations were picked and expanded for further analysis. Clones were analyzed by flow cytometry for AcGFP expression. One AcGFP and one AcGFP-ZIKV capsid clone with similar AcGFP expression were picked to use in future experiments. ZIKV capsid expression was verified by immunoblotting with anti-capsid.

### 2.12. Polyinosinic:Polycytidylic Acid (Poly[I:C]) Stimulation

Mock and stably transduced A459 cells expressing AcGFP alone or AcGFP and ZIKV capsid were seeded in 12-well plates (1 × 10^5^ cells/well) and transfected the next day with 2 µg of Poly(I:C) (InvivoGen) or 2 µg of pCDNA3.1 using TransIT^®^-LT1Transfection Reagent (Mirus Bio, Madison, WI, USA) as per manufacturers’ instructions. Twenty-four hours after Poly(I:C) transfection, cells were washed in PBS and harvested for RNA extraction.

### 2.13. Affinity Purification Mass Spectrometry (AP-MS)

HEK293T cells at 70% confluence in five 100-mm dishes were transfected with 5 μg of plasmid encoding 3xFLAG-tagged ZIKV capsid or 3xFLAG-tagged LacZ as a negative control. Forty-eight hours later, the cells were re-suspended in 4 pellet volumes of NP40 lysis buffer (50 mM Tris pH 8, 100 mM KOAc, 2 mM EDTA, 0.5% NP40, 0.1% Na Deoxycholate, and P8340 protease inhibitor cocktail (Sigma-Aldrich, St. Louis, MO, USA)) and incubated 30 min on ice. FLAG-tagged proteins were recovered from clarified lysates on anti-FLAG resin, and recovered proteins identified as previously described [30].

### 2.14. Co-Immunoprecipitation

Mock and stably transduced A459 cells expressing AcGFP alone or AcGFP and myc-tagged ZIKV capsid (1 × 10^6^) were lysed in 400 mL IP buffer (50 mM Tris-HCL (pH 7.6), 20 mM NaCl, 1 mM MgCL2, 1% Triton X-100, 1× cOmplete™ mini EDTA-free protease inhibitor cocktail (Roche, Indianapolis, USA) and 1× PhosSTOP (Roche) for 15 min at 4 °C with rotation, and then clarified by centrifugation at 14,000 rpm for 10 min. The cleared lysates were incubated with anti-myc magnetic beads (Thermo-Scientific) overnight with rotation at 4 °C. The beads were washed twice with 400 mL IP buffer before bound proteins were eluted by heating at 95 °C for 5 min in 40 μL of 2× Laemmli sample buffer containing 5% β-mercaptoethanol, followed by SDS-PAGE and immunoblotting. Where indicated, A459 cells transiently expressing myc-tagged ZIKV, YFV, DENV, WNV, JEV or SLEV capsids were processed in the same manner at forty-hours post-transfection.

### 2.15. Knockdown of TRIM25

A549 cells (1 × 10^5^ cells/well) seeded into a 12-well plate were transfected the next day with Dicer-substrate siRNA (DsiRNA) targeting TRIM25 (Design ID: hs.Ri.TRIM25.13.1) or a non-targeting DsiRNA control (Integrated DNA technologies, Coralville, IO, USA) at 12 pmol per well using Lipofectamine RNAiMAX reagent (ThermoFisher). The next day, cells were infected with ZIKV (MOI of 3) and 48 h post-infection total cellular RNA was extracted, and media were collected for qRT-PCR and viral plaque assays respectively.

### 2.16. Over-Expression of TRIM25

A549 cells seeded in 6-well plates were transfected when they were 80% confluent with 1 µg of control (pCDNA3.1) or pFlagCMV2-EFP-TRIM25 [43] plasmids, and then 24-h later, were infected with ZIKV (PRVABC-59) at an MOI of 3. Supernatants were collected for quantification by plaque assay at 48 h post-infection. The plasmid pFlagCMV2-EFP-TRIM25 was obtained from Addgene (plasmid # 12449).

### 2.17. GST Pulldown and Immunoblotting

HEK293T cells in 6-well plates were co-transfected with 1 µg of each plasmid construct when they were 80% confluent, as indicated, one well per sample (2 µg of DNA was used for plasmids encoding Flag-tagged viral proteins or substituted with empty vector where absent). Twenty-four hours later, cells were lysed in 1 mL of Triton X-100 lysis buffer (50 mM Tris-HCl pH 7.2, 150 mM NaCl, 1% [vol/vol] Triton X-100, cOmplete™ mini EDTA-free protease inhibitor cocktail (Roche)), followed by two sonication cycles (10-s each). Cell lysates were centrifuged at 14,000 rpm at 4 °C for 15 min in a microfuge before being used for GST-pulldowns. Clarified lysates were mixed with 50 µL of glutathione Sepharose 4B resin (GE Healthcare, Mississauga, ON, Canada) that had pre-equilibrated with Triton X-100 lysis buffer, and were then incubated with rotation for 3 to 4 h at 4 °C. The beads were washed four times with ice-cold Triton X-100 lysis buffer and then bound proteins were eluted by boiling in Laemmli sample buffer for 10 min before SDS-PAGE, transferred to PVDF membrane and followed by immunoblotting. Proteins on the membranes were detected with anti-V5 monoclonal (Fisher), anti-Flag-M2 monoclonal (Sigma-Aldrich, St. Louis, MO, USA), anti-HA-HRP monoclonal (GenScript, Piscataway, NJ, USA), and anti-GST polyclonal (Sigma-Aldrich, St. Louis, MO, USA). Primary antibodies were detected with polyclonal goat anti-mouse (Thermo-Fisher, Ottawa, ON, Canada) or anti-rabbit-HRP (BioRad, Hercules, CA, USA) secondary antibodies and visualized by chemiluminescence using Pierce^®^ ECL Western blotting substrate (Thermo Scientific, Ottawa, ON, Canada) and a ChemiDoc imager (Bio-Rad, Hercules, CA, USA).

## 3. Results

### 3.1. Global Gene Expression Changes in Capsid-Expressing Cells

**Figure 1:**
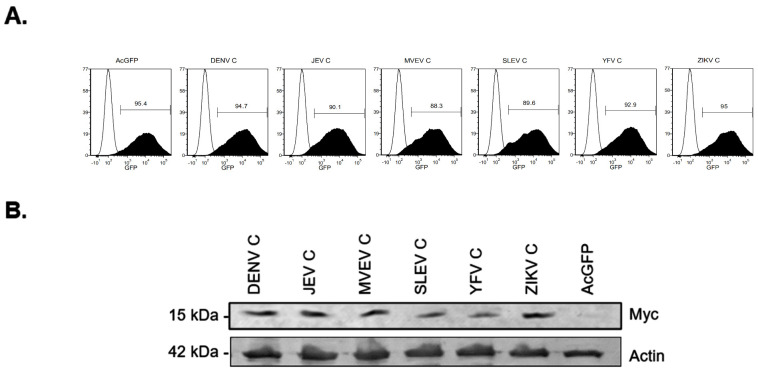
Expression of myc-tagged capsid proteins. A549 cells were transduced with lentiviral vectors expressing AcGFP alone or AcGFP and myc-tagged capsid proteins. Cells were collected at 48 h post-transduction for analysis by flow cytometry and immunoblotting. (**A**) GFP expression of transduced cells was measured by flow cytometry. Values shown are % GFP-positive cells relative to mock. (**B**) Cell lysates from duplicate samples were analyzed by immunoblotting with anti-Myc and anti-Actin.

**Figure 2:**
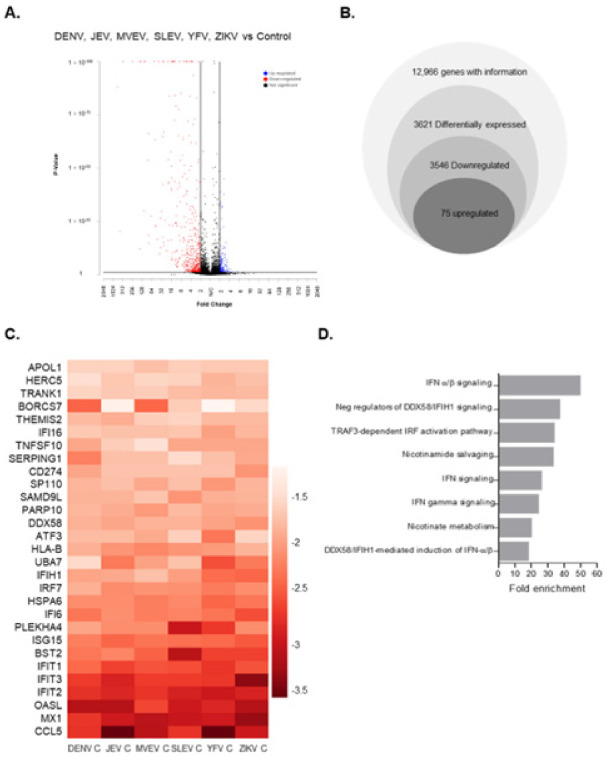
Global gene expression changes in capsid-expressing cells. A549 cells were transduced with lentiviral vectors expressing AcGFP alone or AcGFP and myc-tagged capsid proteins. Cells were collected at 48 h post-transduction, and then total RNA was extracted and processed for RNA-sequencing. Shown are the global gene expression changes common amongst all capsid-expressing cells relative to control (AcGFP). (**A**) Volcano plot displaying differentially expressed genes. Differentially expressed genes with >2-fold changes and with a *p*-value ≤ 0.01 are shown. Blue: upregulated; red: downregulated; grey: not significant. (**B**) Total common gene expression changes in capsid-expressing cells. All genes with a *p*-value of ≤ 0.01 are included. In total, 3621 genes were differentially expressed (>2-fold change). (**C**) Top 30 downregulated genes in cells transduced with flavivirus capsids. Each panel in the heat map represents a particular gene, and the intensity of the colour represents fold change relative to control (all values underwent log 10 transformation). (**D**) Gene ontology enrichment analysis of common differentially expressed genes (DEGs) among capsids. Biological pathways were determined by individually submitting DEG lists of downregulated genes to the online bioinformatics server (geneontology.org) using the annotation data set “Reactome pathways”. Shown are the top eight enriched pathways.

Multiple studies have investigated how ZIKV infection affects the transcriptomes of human microglia, fibroblasts, monocyte-derived macrophages, astrocytes and dendritic cells [44,45,46,47]. Data from these studies indicate that ZIKV infection induces massive transcriptional changes in host cells; however, it is not clear if/how individual viral proteins affect these processes. Here, RNA-sequencing was used to assess the global transcriptome changes in cells expressing flavivirus capsid proteins which are known to enter the nuclei of infected cells. Lentiviruses encoding AcGFP and myc-tagged capsid proteins from Dengue virus (DENV), Japanese encephalitis virus (JEV), Murray Valley encephalitis virus (MVEV), St. Louis encephalitis virus (SLEV), Yellow Fever virus (YFV) and Zika virus (ZIKV) capsid proteins have been described [30]. A549 cells were transduced with lentiviruses encoding myc-tagged capsid proteins or AcGFP alone using an MOT of 10. Forty-eight hours post-transduction all samples were approximately 90% AcGFP positive, indicating that most cells had been transduced and were therefore expressing capsid proteins (Figure 1A). Immunoblotting confirmed expression of the capsid proteins of correct sizes in transduced cells (Figure 1B). Next, total RNA extracted from duplicate samples and differentially expressed genes (DEGs) in capsid-expressing cells were identified by RNA-seq. Analyses revealed 3546 downregulated and 75 upregulated DEGs shared among cells expressing different capsid proteins compared to cells expressing AcGFP alone (Fold change ≥ 2, *p*-value ≤ 0.01) (Figure 2A,B). The top 20 down- and upregulated genes are listed in Table 1 and Table 2, respectively. Strikingly, the DEGs that were downregulated the most (≥10-fold) as a result of flavivirus capsid protein expression, are involved in the IFN response (e.g., *CCL5, MX1, OASL, IFIT2*) (Figure 2C). Gene enrichment analysis was performed on all genes that were downregulated ≥10-fold, and the top biological process significantly enriched in this group was “IFNα/β signaling” (Figure 2D).

### 3.2. Validation of Transcriptomic Changes Observed in RNA-Seq Analyses

**Figure 3:**
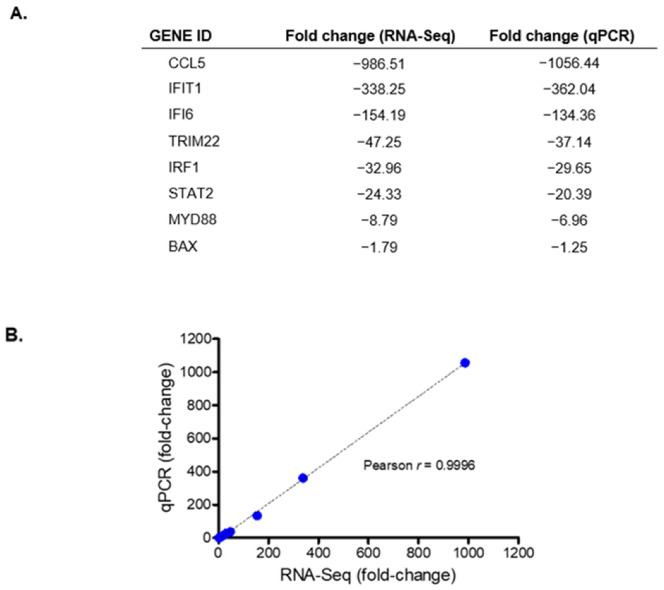
Validation of RNA-Sequencing. (**A**) Shown is the fold change of select genes that were generated from RNA-seq and qPCR for DENV capsid-expressing cells. (**B**) The fold-change obtained from RNA-Seq and qPCR were plotted with a line of best fit.

To validate the transcriptomic changes identified from RNA-sequencing, cDNAs were prepared from the same RNA samples used for the deep sequencing. As most of the DEGs identified in the RNA-seq analyses were downregulated, eight downregulated genes with fold changes between 1 and 1000 were selected for validation by qPCR, which was performed with primers that had the same sequences used for the RNA-sequencing. The gene expression changes as measured by qRT-PCR and those derived from RNA-sequencing were very similar, as indicated by fact that the correlation coefficient between the select genes (Pearson’s r) was 0.9996 (Figure 3).

### 3.3. Cells Transduced with Lentiviruses Encoding ZIKV Capsid Suppress IFN-β and Are Susceptible to Viral Infection

**Figure 4:**
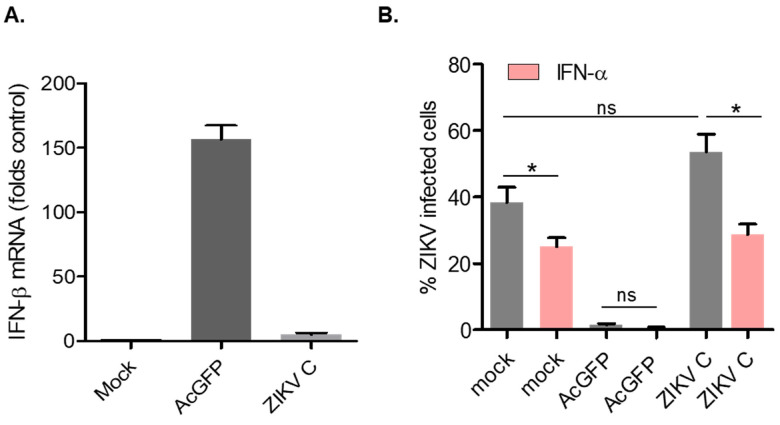
ZIKV capsid expressing cells suppress IFN-β and are highly susceptible to infection. A549 cells were transduced with lentivirus expressing AcGFP or AcGFP-Myc-ZIKVC (MOT of 10). (**A**) AcGFP expressing cells have high levels of IFN-β mRNA. Cells were collected at 48 h post-transduction and the levels of IFN-β mRNA in mock, AcGFP or ZIKV C-expressing cells were determined by qRT-PCR. (**B**) ZIKV C expressing cells are permissive to ZIKV infection but are sensitive to pre-treatment with IFN-α. Transduced cells were infected with ZIKV at an MOI of 3 (grey bars) or pre-treated with IFN-α prior to ZIKV infection (pink bars). All cells were collected 48 h post-infection and stained with anti-ZIKV NS1 antibody. Only the GFP-positive cells were included in the analysis. Average results from 3 independent experiments are shown. Results of statistical analyses (*t*-test) are indicated (* *p* ≤ 0.05). ns, not significant.

Previous studies have shown that lentiviral transduction can trigger a transient type I IFN response, both in vitro and in vivo. For example, in dendritic cells, lentivirus transduction leads to activation of toll-like receptors and production of IFN-β, IL-23 and other ISGs [48]. Similarly, administration of lentiviruses to mice induces rapid but transient expression of *IFN-a* and *IFN-b* [49].To determine if capsid-encoding lentiviruses were triggering a type I IFN response in A549 cells, RNA from transduced cells expressing AcGFP alone (control) or AcGFP and Myc-ZIKV capsid was subjected to qRT-PCR to quantify relative levels of *IFN-β* transcripts. Interestingly, in cells expressing AcGFP alone, levels of *IFN-β* were increased approximately 150-fold compared to mock-transduced cells (Figure 4A). Conversely, induction of *IFN-β* in cells expressing ZIKV capsid was only four-fold higher than in non-transduced cells. These data suggest that ZIKV capsid expression suppresses induction of *IFN-β* during lentiviral transduction.

Next, we assessed whether transduced cells expressing ZIKV capsid protein were more susceptible to infection because of their ability to suppress expression of *IFN-β*. A549 cells were transduced with lentiviruses encoding AcGFP or AcGFP and Myc-ZIKV capsid, and then infected with ZIKV for 48 h, followed by flow cytometry analysis using antibodies to ZIKV NS1 protein. Cells transduced with lentivirus expressing AcGFP alone were resistant to ZIKV infection, whereas capsid-expressing cells were permissive to infection (Figure 4B). Of note, ZIKV capsid-expressing cells appeared to be even more susceptible to ZIKV infection (>50% NS1 positive) than mock-transduced cells (<40% NS1 positive), albeit this difference was not statistically significant. Pretreatment of cells with IFN-α prior to ZIKV infection reduced infection in all samples (pink bars Figure 4B), suggesting that capsid-expressing cells are still sensitive to IFN-α and do not actively subvert type I IFN signaling.

### 3.4. ZIKV Capsid-Expressing Cells Subvert IFN Induction

**Figure 5:**
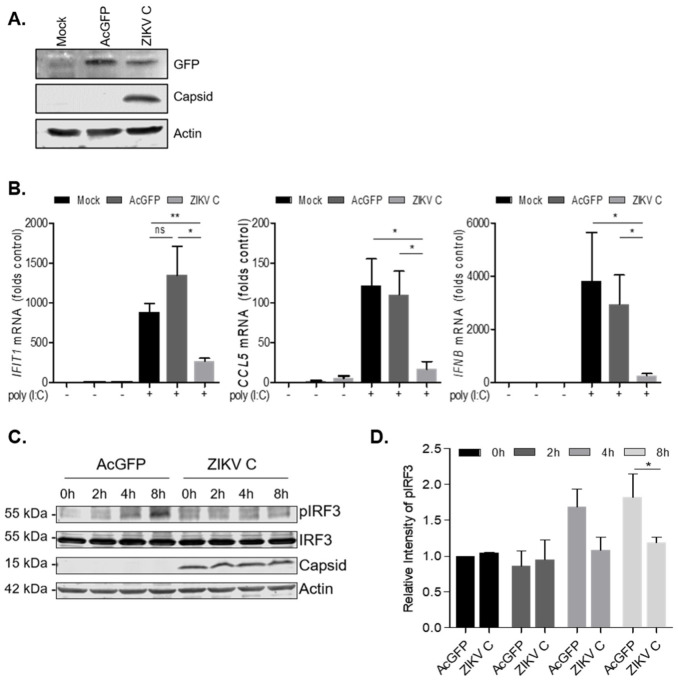
ZIKV capsid-expressing cells suppress IFN induction. (**A**) Immunoblot blot of GFP, ZIKV capsid and actin in cell lysates from AcGFP or AcGFP-ZIKV capsid-expressing stable cell lines. (**B**) Expression of select ISGs in AcGFP and ZIKV C-expressing cells. Cells were transfected with poly (I:C) for 24 h, total RNA was extracted and used to quantify IFIT1, CCL5, IFN-β gene expression by qRT-PCR. Shown are normalized values relative to mock cells (control). Data were from three independent experiments. (**C**) Time course of p-IRF3 in AcGFP or ZIKV C stables following poly (I:C) transfection. Western blot with antibodies that detect pIRF3, IRF3, ZIKV capsid, and actin are shown. (**D**). Mean relative intensity of Western blots based on three independent experiments. Results of statistical analyses (*t*-test) are indicated (* *p* ≤ 0.05) (**, *p* ≤ 0.01). ns, not significant.

To further examine how flavivirus capsid expression affects IFN induction, we created stable cell lines expressing AcGFP or AcGFP and Myc-ZIKV capsid (Figure 5A). QRT-PCR analyses of RNA extracted from these cell lines revealed that basal levels of ISG expression was comparable to mock-treated cells, and thus we anticipated that they would be suitable for subsequent IFN induction studies. Compared to mock transduced and AcGFP-expressing cells, stably transduced cells expressing ZIKV capsid exhibited significantly lower levels of *IFIT1, CCL5* and *IFN-*β transcripts following poly (I:C) transfection (Figure 5B). Given that IRF-3 is required for evocation of type I IFN responses both in vitro and in vivo [50], we next determined whether capsid protein expression affected the activation of this antiviral transcription factor. Lysates from stably transduced cells expressing either AcGFP or AcGFP and Myc-ZIKV capsid after poly (I:C) transfection at several timepoints were analyzed by immunoblotting using antibodies for total and phospho-IRF3. Data in Figure 5C,D show that levels of active (phosphorylated) IRF3 were lower in cells expressing ZIKV capsid protein.

### 3.5. ZIKV Capsid Interacts with TRIM25 and Suppresses RIG-I Ubiquitination

**Figure 6:**
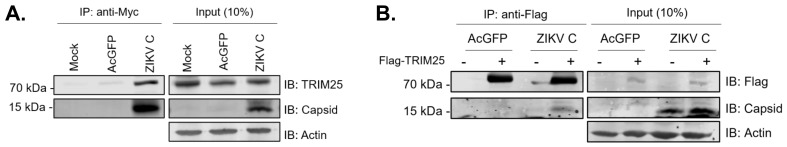
ZIKV capsid interacts with TRIM25. (**A**) Myc-tagged ZIKV capsid-expressing cells were lysed and co-immunoprecipitation was performed using anti-Myc beads. Immunoblots were probed for TRIM25, capsid and actin. (**B**) Flag-TRIM25-expressing cells were lysed and co-immunoprecipitation was performed using anti-Flag beads. Immunoblots were probed for Flag, capsid and actin. Each experiment in panels A and B was conducted at least three times.

**Figure 7:**
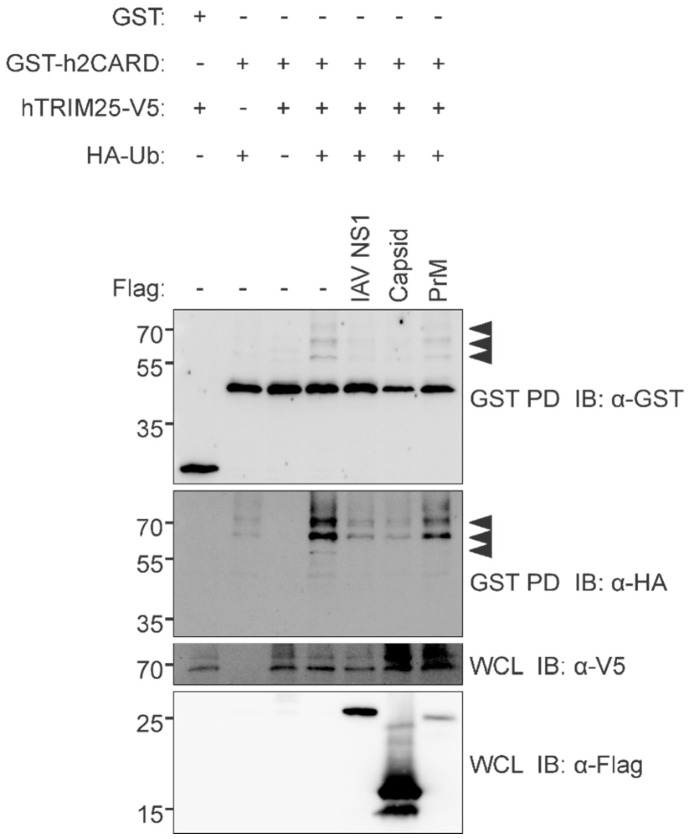
ZIKV capsid inhibits TRIM25 from ubiquitinating RIG-I. HEK293T cells were transfected with GST-tagged human RIG-I CARD domains (GST-h2CARD) or empty GST vector, together with HA-tagged ubiquitin (HA-Ub), V5-tagged human TRIM25 (hTRIM25-V5), and the indicated Flag-tagged proteins. Clarified whole cell lysates (WCL) were subjected to GST pulldown (GST PD), followed by immunoblotting (IB) with anti-GST, anti-HA, anti-V5, and anti-Flag antibodies. Influenza A virus (IAV) NS1 serves as a positive control for ubiquitination inhibition.

To further understand how ZIKV capsid impairs IFN induction, we performed affinity purification with ZIKV capsid coupled to mass spectrometry (AP-MS) and identified TRIM25 as a binding partner (Table 3). TRIM25 is an E3 ubiquitin ligase that regulates the function of RIG-I by K63-linked polyubiquitination [51]. Upon recognition of viral RNA, RIG-I undergoes a conformational change that exposes the CARD domain. RIG-I then homo-oligomerizes on the viral RNA, after which it is polyubiquitinated by TRIM25. Ubiquitination of RIG-I facilitates its interaction with MAVS, which ultimately leads to activation of IRF3 and induction of IFN [52]. To validate interaction of ZIKV capsid with TRIM25, lysates from A549 cells expressing AcGFP and Myc-ZIKV capsid or AcGFP alone (control) were subjected to co-immunoprecipitation with anti-myc followed by immunoblotting with anti-TRIM25 and anti-ZIKV capsid antibodies (Figure 6A). Anti-Flag pulldowns from lysates of A549 cells expressing AcGFP and Myc-ZIKV capsid or AcGFP (control) with or without Flag-TRIM25 confirmed that ZIKV capsid formed a stable complex with TRIM25 (Figure 6B). Next, to test if ZIKV capsid expression affected ubiquitination of RIG-I, HEK 293T cells transfected with plasmids encoding ZIKV capsid or prM proteins and GST-tagged RIG-I CARD domains or empty vector together with HA-tagged ubiquitin and V5-tagged TRIM25 were subjected to GST pulldowns followed by immunoblotting with antibodies to HA, V5 or ubiquitin. The data shown in Figure 7 show that ZIKV capsid, but not M protein, inhibits ubiquitination of RIG-I CARD domains to a similar extent as that observed with the positive control influenza A virus NS1. Given that the capsid proteins of DENV, YFV, MVEV, JEV and SLEV also form stable complexes with TRIM25 (Supplemental Figure S1), blocking activating ubiquitination of RIG-I may be a common mechanism used by flaviviruses to dampen IFN induction.

### 3.6. TRIM25 Is a Viral Restriction Factor

Many viruses induce degradation of host cell-encoded viral restriction factors as a way to impair the innate immune response. To test whether TRIM25 is targeted for degradation during ZIKV infection, immunoblot analysis was performed on infected A549 cell lysates prepared at 24 and 48 h post-infection. These experiments show that levels of TRIM25 protein were not depleted during ZIKV infection (Supplementary Figure S2).

**Figure 8:**
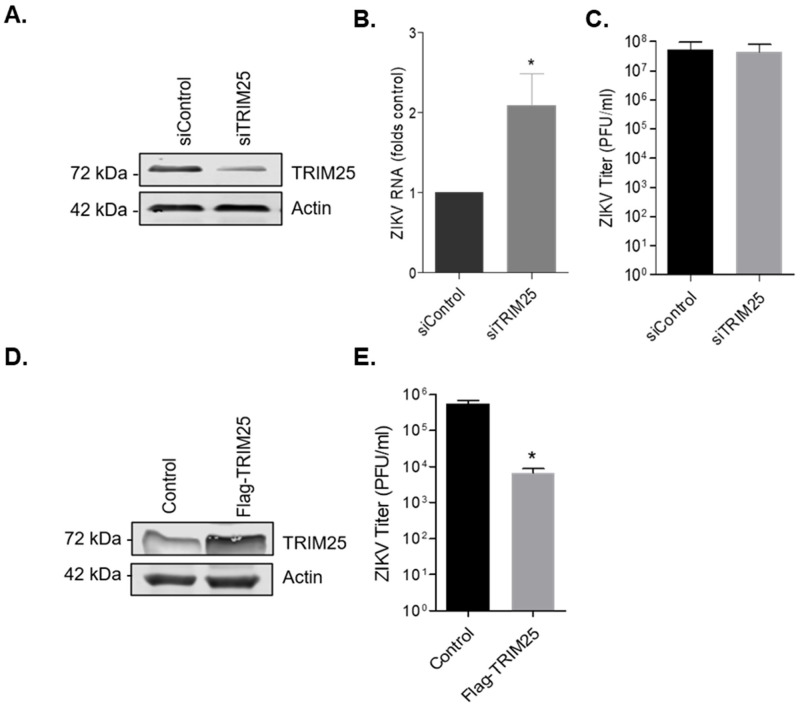
TRIM25 is a viral restriction factor. (**A**) A549 cells were transfected with siNon-targeting control (siControl) or siTRIM25 for 48 h, followed by infection with ZIKV (MOI of 3). Western blots of cell lysates 48 h post-transfection with desired siRNA and probed with antibodies for TRIM25 and actin. (**B**) Cellular RNA was extracted at 48 h post-infection, and qRT-PCR using ZIKV specific primers was performed. (**C**) Supernatant was collected for quantification by plaque assay. (**D**) A549 cells were transfected with pCDNA3.1 or pFlag-TRIM25 for 48 h, followed by infection with ZIKV (MOI of 3). Shown are the Western blots of cell lysates 24 h post-transfection and probed with antibodies for TRIM25 and actin. (**E**) Supernatant was collected for quantification by plaque assay. The average values from three independent experiments are shown. All data were subject to *t*-test analyses (* *p* ≤ 0.05).

To further analyze the role of TRIM25 during ZIKV infection, we utilized dicer-substrate siRNA (DsiRNA) targeting TRIM25 (siTRIM25) or a non-targeting DsiRNA control (siControl). A549 cells were transfected with DsiRNAs for 48 h, after which cell lysates were analyzed for TRIM25 protein levels by immunoblotting (Figure 8A). On average, the level of TRIM25 protein was reduced by approximately 75% following siTRIM25 transfection. Next, cells were infected with ZIKV and 48 h later, total cellular RNA was extracted, and cell media were collected for qRT-PCR and viral plaque assays respectively. Reduction of TRIM25 expression resulted in a small (approximately 2-fold) but statistically significant increase in ZIKV replication, as determined by qRT-PCR using specific primers to ZIKV genomic RNA (Figure 8B), however, viral titers were not affected (Figure 8C). In contrast, ectopic expression of TRIM25 resulted in almost 100-fold drop in ZIKV titers (Figure 8D,E). These data indicate that TRIM25 is a restriction factor for ZIKV whose function may be abrogated by capsid-mediated sequestration during infection.

## 4. Discussion

One of the main functions of flavivirus capsid proteins is to protect viral genomes during cell entry and exit. However, mounting evidence suggests that these viral proteins can also subvert host antiviral defense systems. Given that capsid proteins are the first viral proteins made during infection, this presents a clear advantage for flaviviruses to inhibit antiviral signaling even before nonstructural viral proteins exert their effects on the IFN system. YFV and other flavivirus capsid proteins have been reported to suppress the RNA interference pathway by binding to dsRNA [31]. While the role of RNA interference in combatting viral infection in mammals has been controversial, more recent data suggest that it may indeed be an effective arm of the innate immune response (reviewed in [53]). In another study, DEAD box helicase 3 X-linked (DDX3X) was identified as a DENV capsid-binding protein [32]. This helicase is important for induction of IFN through its interaction with multiple antiviral signaling molecules, including MAVS, and the kinases TBK1 and IKK-e [54,55,56,57]. As such, capsid-mediated sequestration of this RNA helicase could potentially affect the innate immune response by preventing binding of DDX3X with one or more partners that function in antiviral signaling [32]. Interestingly, influenza A virus NS1 protein, which has well-defined roles in suppressing the innate immune response, including through interaction with TRIM25 [58], shares some structural similarity with DENV capsid [59]. Accordingly, it was postulated that DENV capsid may function similarly to dampen the IFN response.

The reports cited above describe multiple mechanisms by which flavivirus capsid proteins may interfere with host antiviral defense systems through interactions with cytoplasmic proteins. However, given that a pool of capsid protein localizes to the nuclei during flavivirus infection [34,60,61], it is tempting to speculate that host transcription could be altered in a way that affects the innate immune response. Indeed, gene ontology enrichment analyses revealed that expression of flavivirus capsid proteins from DENV, JEV, SLEV, MVEV, YFV, and ZIKV suppressed expression of host genes involved in “Interferon-α/β signaling”. Subsequent analyses showed that ZIKV capsid interacts with the E3 ligase TRIM25, and inhibits ubiquitination of RIG-I, which is important for IFN induction. All of the other flavivirus capsid proteins used in this study also formed stable complexes with TRIM25, suggesting that capsid-mediated inhibition of TRIM25 is a common strategy used by flaviviruses to dampen the IFN response. While this can explain why genes involved in IFN signaling are suppressed in capsid-expressing cells, it is not likely related to the nuclear pool of capsid protein. The vast majority of TRIM25 localizes exclusively to the cytoplasm, including during its interaction with another viral protein, influenza NS1, which also suppresses TRIM25-mediated ubiquitination of RIG-I [62]. At this point it cannot be ruled out the nuclear pool of flavivirus capsid proteins affects host gene expression through as yet other mechanisms, including disruption of nucleosomes by interacting with histone proteins [34].

Ectopic expression of TRIM25 reduced ZIKV titers by almost 100-fold, indicating that TRIM25 is a viral restriction factor. The observation that knockdown of TRIM25 did not dramatically increase ZIKV replication may be because interaction of this E3 ubiquitin ligase with capsid protein, which is very abundant in infected cells, effectively blocks its function such that siRNA-mediated knockdown does not have any additional effect. However, it is also possible that capsid employs TRIM25-independent mechanisms to subvert the IFN response. Indeed, we and another group showed that ZIKV capsid interacts with protein phosphatase-1 [30,63] which is known to activate RIG-I and MDA-5 [19]. Finally, using an integrated proteomics approach, Scaturro et al. identified TRIM26 as a ZIKV capsid-binding protein in the cytoplasm [63]. Unlike TRIM25, which promotes IFN induction, TRIM26 appears to do the opposite by ubiquitinating IRF3 which is then degraded [64,65]. As such, the biological relevance of capsid interaction with TRIM26 during ZIKV infection is not clear.

Collectively, our findings reveal a novel, previously unrecognized role for flavivirus capsids in subverting the innate immune response before nonstructural proteins are produced. Future studies will focus on elucidating how interaction of ZIKV, and other flavivirus capsid proteins with TRIM25 and other immune modulators in the cytoplasm and nucleus, affect antiviral signaling.

## Figures and Tables

**Table 1:** List of top 20 downregulated differentially expressed genes (*p*-value ≤ 0.01).

Gene ID	Description	Fold Change
CCL5	C-C Motif Chemokine Ligand 5	−1363.33
MX1	MX Dynamin Like GTPase 1	−1055.14
OASL	2’-5’-Oligoadenylate Synthetase Like	−956.56
IFIT2	Interferon Induced Protein with Tetratricopeptide Repeats 2	−718.98
IFIT3	Interferon Induced Protein with Tetratricopeptide Repeats 3	−694.00
IFIT1	Interferon Induced Protein with Tetratricopeptide Repeats 1	−363.25
BST2	Bone Marrow Stromal Cell Antigen 2	−352.47
ISG15	ISG15 Ubiquitin-Like Modifier	−221.56
PLEKHA4	Pleckstrin Homology Domain Containing A4	−212.54
IFI6	Interferon Alpha Inducible Protein 6	−185.33
HSPA6	Heat Shock Protein Family A (Hsp70) Member 6	−159.88
IRF7	Interferon Regulatory Factor 7	−117.75
IFIH1	Interferon Induced with Helicase C Domain 1	−111.11
UBA7	Ubiquitin Like Modifier Activating Enzyme 7	−108.42
HLA-B	Major Histocompatibility Complex, Class I, B	−95.93
ATF3	Activating Transcription Factor 3	−88.65
DDX58	DExD/H-Box Helicase 58	−79.13
PARP10	Poly (ADP-Ribose) Polymerase Family Member 10	−73.80
SAMD9L	Sterile Alpha Masotif Domain Containing 9 Like	−68.68
SP110	SP110 Nuclear Body Protein	−67.85

**Table 2:** List of top 20 upregulated differentially expressed genes (*p*-value ≤ 0.01).

Gene ID	Description	Fold Change
PRELID2	PRELI Domain Containing 2	14.96
ATP5MD	ATP Synthase Membrane Subunit DAPIT	11.83
SEC31A	SEC31 Homolog A, COPII Coat Complex Component	10.48
TXNDC5	Thioredoxin Domain Containing 5	10.13
ANKS4B	Ankyrin Repeat and Sterile Alpha Motif Domain Containing 4B	8.24
TBC1D22A	TBC1 Domain Family Member 22A	5.22
ITGB3BP	Integrin Subunit Beta 3 Binding Protein	4.80
SCARNA3	Small Cajal Body-Specific RNA 3	4.20
DDC	Dopa Decarboxylase	4.20
PIK3C2A	Phosphatidylinositol-4-Phosphate 3-Kinase Catalytic Subunit Type 2 Alpha	3.99
CENPE	Centromere Protein E	3.96
PLPPR1	Phospholipid Phosphatase Related 1	3.80
TMEM107	Transmembrane Protein 107	3.70
PMS2	PMS1 Homolog 2, Mismatch Repair System Component	3.63
CDC25C	Cell Division Cycle 25C	3.61
EFCAB10	EF-Hand Calcium Binding Domain 10	3.58
NUP43	Nucleoporin 43	3.25
NONO	Non-POU Domain Containing Octamer Binding	3.23
PDK3	Pyruvate Dehydrogenase Kinase 3	3.15
HMMR	Hyaluronan Mediated Motility Receptor	3.13

**Table 3:** Affinity Purification Mass Spectrometry performed with ZIKV capsid protein reveals an interaction with the host protein TRIM25. The top twelve other high confidence hits based on spectral counts from two independent experiments are also shown.

		Spectral	Counts	
Protein ID	LacZ	Capsid (Exp 1)	Capsid (Exp 2)	Crapome Average
Q99733|NP1L4	2	39	37	3.1
Q01105|SET	0	26	24	5.4
Q9BY44|EIF2A	0	16	21	2.9
6P158|DHX57	1	15	15	2
Q14258|TRIM25	0	16	13	1.9
Q96GA3|LTV1	1	13	14	2.7
Q9Y6M1|IF2B2	1	13	13	3.4
Q8WU90|ZC3HF	1	13	12	3.1
O15355|PPM1G	0	15	10	2
Q12797|ASPH	0	12	12	1.5
Q9NX00|TM160	0	10	12	1
Q99543|DNJC2	0	13	5	1.3
Q13144|EI2BE	0	9	9	1.4

## Data Availability

Not applicable.

## Supplementary Materials

The following supporting information can be downloaded at: https://www.mdpi.com/article/10.3390/v14050968/s1, Figure S1: Flavivirus capsids interact with TRIM25; Figure S2: TRIM25 is not degraded during ZIKV infection; Table S1: List of primers used in this study.
