# Supplementary material for: Flavivirus Capsid Proteins Inhibit the Interferon Response
Source: Viruses. 2022 May 5;14(5):968. doi: 10.3390/v14050968 (PMC9146811; doi:10.3390/v14050968)
Supplement: Supplementary file 1 [file viruses-14-00968-s001.zip › viruses-1623080-supplementary.pdf]

viruses

MDPI

Supplementary Material

Table S1. List of primers used in this study.

Target	Primer sequence (5′-> 3′)	Source
IFIT1 Fw	TGCAGAACGGCTGCCTAATTT	Sequence from AmpliSeq (Thermo Fisher) manufactured by IDT
IFIT1 Rv	CAA-GACTCTGTTTTCTAAATCAGGCATT	Sequence from AmpliSeq (Thermo Fisher) manufactured by IDT
IFI6 Fw	CGCTGCTGTGCCCTCTAT	Sequence from AmpliSeq (Thermo Fisher) manufactured by IDT
IFI6 Rv	GCAAGTGAAGAGCAGCAGGTA	Sequence from AmpliSeq (Thermo Fisher) manufactured by IDT
CCL5 Fw	CTCGCTGTCATCCTCATTGCT	Sequence from AmpliSeq (Thermo Fisher) manufactured by IDT
CCL5 Rv	GCACTTGCCACTGGTGTAGA	Sequence from AmpliSeq (Thermo Fisher) manufactured by IDT
TRIM22 Fw	CTCTGGCTTGGTGAGTGAATCT	Sequence from AmpliSeq (Thermo Fisher) manufactured by IDT
TRIM22 Rv	CCCTTGGCTTCCTTCTGTCTT	Sequence from AmpliSeq (Thermo Fisher) manufactured by IDT
IRF1 Fw	CCAGGCTACATGCAGGACTT	Sequence from AmpliSeq (Thermo Fisher) manufactured by IDT
IRF1 Rv	GGGTGACACCTGGAAGTTGTA	Sequence from AmpliSeq (Thermo Fisher) manufactured by IDT
STAT2 Fw	ATTGACCACGGGTTGGAACA	Sequence from AmpliSeq (Thermo Fisher) manufactured by IDT
STAT2 Rv	CGTAGGTCCACCCCTTTGG	Sequence from AmpliSeq (Thermo Fisher) manufactured by IDT
MYD88 Fw	CGGGCATCACCACACTTGAT	Sequence from AmpliSeq (Thermo Fisher) manufactured by IDT
MYD88 Rv	CAGACACACACAACTTCAG-TCGATA	Sequence from AmpliSeq (Thermo Fisher) manufactured by IDT
BAX Fw	CCAGCTGTGAGCAGATCATGAA	Sequence from AmpliSeq (Thermo Fisher) manufactured by IDT
BAX Rv	GAGACACTCGCTCAGCTTCT	Sequence from AmpliSeq (Thermo Fisher) manufactured by IDT
IFNB1 Fw	TGTGCCTGGACCATAGTCAGA	Sequence from AmpliSeq (Thermo Fisher) manufactured by IDT
IFNB1 Rv	AACAGCATCTGCTGGTTGAAGA	Sequence from AmpliSeq (Thermo Fisher) manufactured by IDT
B-actin Fw	CCT GGC ACC CAG CAC AAT	Airo Am, Urbanowski MD, et al. Virol-ogy, 2018;516:147-57
B-actin Rv	GCC GAT CCA CAC GGA GTA CT	Airo Am, Urbanowski MD, et al. Virol-ogy, 2018;516:147-57

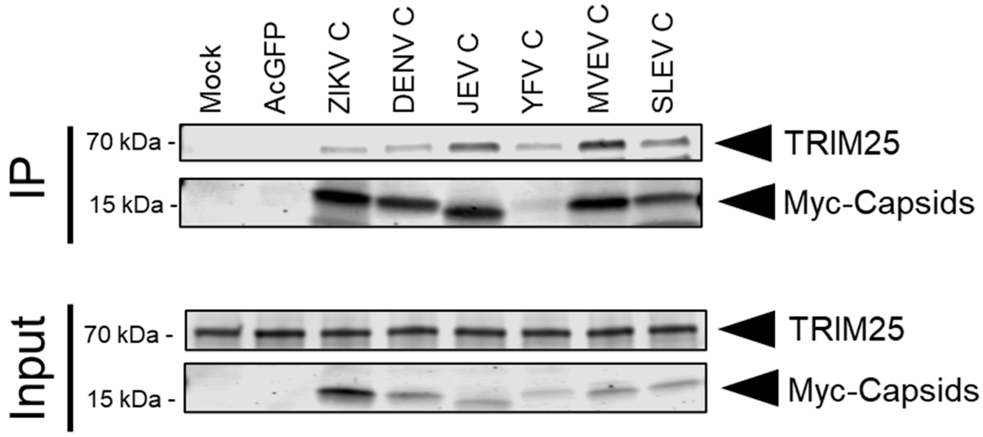

Figure S1. Flavivirus capsids interact with TRIM25. A549 cells expressing Myc-tagged flavivirus capsids were lysed and co-immunoprecipitation was performed using anti-Myc beads. Immunoblots were probed for TRIM25 and Myc.

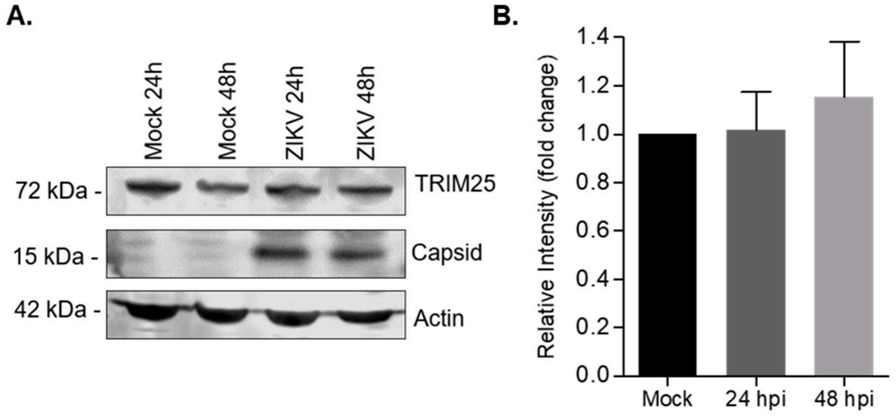

Figure S2. TRIM25 is not degraded during ZIKV infection. A549 cells were infected with ZIKV (MOI of 3) and cell lysates were collected 24 and 48 hours post-infection. **(A)** Western blot of cell lysates from Mock or ZIKV-infected cells at 24- or 48-hours post-infection. Antibodies specific to TRIM25, ZIKV capsid or Actin were used. **(B)** Relative intensity (fold change) of TRIM25 levels by immunoblotting as determined from 3 independent-experiments.
